# Functional Analyses of Transcription Factor Binding Sites that Differ between Present-Day and Archaic Humans

**DOI:** 10.1093/molbev/msv215

**Published:** 2015-10-09

**Authors:** Sven Weyer, Svante Pääbo

**Affiliations:** ^1^Department of Evolutionary Genetics, Max-Planck-Institute for Evolutionary Anthropology, Leipzig, Germany

**Keywords:** transcription factor binding site, human evolution, Neandertal, modern humans, single nucleotide changes

## Abstract

We analyze 25 previously identified transcription factor binding sites that carry DNA sequence changes that are present in all or nearly all present-day humans, yet occur in the ancestral state in Neandertals and Denisovans, the closest evolutionary relatives of humans. When the ancestral and derived forms of the transcription factor binding sites are tested using reporter constructs in 3 neuronal cell lines, the activity of 12 of the derived versions of transcription factor binding sites differ from the respective ancestral variants. This suggests that the majority of this class of evolutionary differences between modern humans and Neandertals may affect gene expression in at least some tissue or cell type.

## Introduction

The closest evolutionary relatives of present-day humans are the Neandertals and Denisovans, two extinct hominins that lived in western Eurasia and Asia, respectively, and shared a common ancestral population with modern humans about 550,000–765,000 years ago (Prüfer et al. 2014). An interesting question is the extent to which these extinct hominins differed in their biology and cognition from modern humans, and if such differences may underlie the dramatic cultural, technological, and demographic development that has characterized the evolution of modern humans (Paabo 2014).

Recently, the genome of a Neandertal individual has been sequenced to high quality (Prüfer et al. 2014). This genome sequence as well as a genome sequence previously generated from a Denisovan individual (Meyer et al. 2012) cover approximately two-thirds of the genome to which short DNA sequences can be mapped on average 42-fold and 30-fold, respectively. Together they make it possible to identify positions in the genome where the majority of present-day humans carry derived alleles, that is, alleles that differ from the apes, which are not seen in the Neandertal and Denisovan genomes. Because the 2 archaic genomes derive from populations that separated from each other some 381,000–473,000 years ago (although limited gene flow subsequently occurred between them) (Prüfer et al. 2014), such derived alleles are unlikely to have been prevalent in the 2 archaic groups. Instead, most of them result from new mutations or alleles that were present at low frequency in the ancestral population of the two archaic groups and modern humans and have since risen to high frequency or fixation among modern humans. These alleles are potentially interesting because some of them may influence biological traits unique to modern humans relative to other, extinct hominin groups. However, it is possible that the majority of these alleles have no biological effects, or effects that are too small to be of biological relevance. A crucial question is therefore if biological effects can be detected in this class of alleles, that is, if some derived alleles that occur at high frequency in present-day humans have effects that differ from the ancestral alleles seen in the Neandertal and Denisovan genomes.

Here we analyze 50 ancestral and derived allelic variants of 25 transcription factor binding sites (TFBSs) with respect to their ability to drive transcription from a reporter construct in 3 neuronal cell lines. We find differences between the two allelic forms in approximately half of them.

## Results

A total of 145,159 single nucleotide changes and small insertions or deletions where the Neandertal and Denisovan genomes both carry only ancestral alleles, that is, alleles inferred to have occurred in the common ancestor shared with chimpanzees, but where 90% or more of present-day humans carry derived variants have been previously identified (Prüfer et al. 2014). Among these variants, which have thus risen to high frequency or fixation in modern humans since their separation from archaic hominins, Prüfer et al. (2014) identified 14,497 that potentially affect “regulatory features” as defined by the Variant Effect Predictor (McLaren et al. 2010) in “Ensemble” based on features such as transcription factor binding, histone modifications, and chromatin accessibility (Flicek et al. 2014). They noted that 139 of these affect TFBSs as defined by the JASPAR data base (Mathelier et al. 2014), and that 25 of them affect positions in the TFBSs that may be important for their ability to bind transcriptional regulators (*s*core > 1.5 according to Stormo 2000; see supplementary tables S18.1 and 18.3 in Prüfer et al. 2014). At 6 of these sites, all present-day humans investigated to date carry the derived variant, while at the remaining 19 sites the ancestral variant is present in less than 10% of present-day humans (supplementary table S1, Supplementary Material online). For 20 of the sites there is direct evidence that they bind transcription factors in neurons or neuronal stem cells (supplementary table S2, Supplementary Material online).

**Fig. 1.**
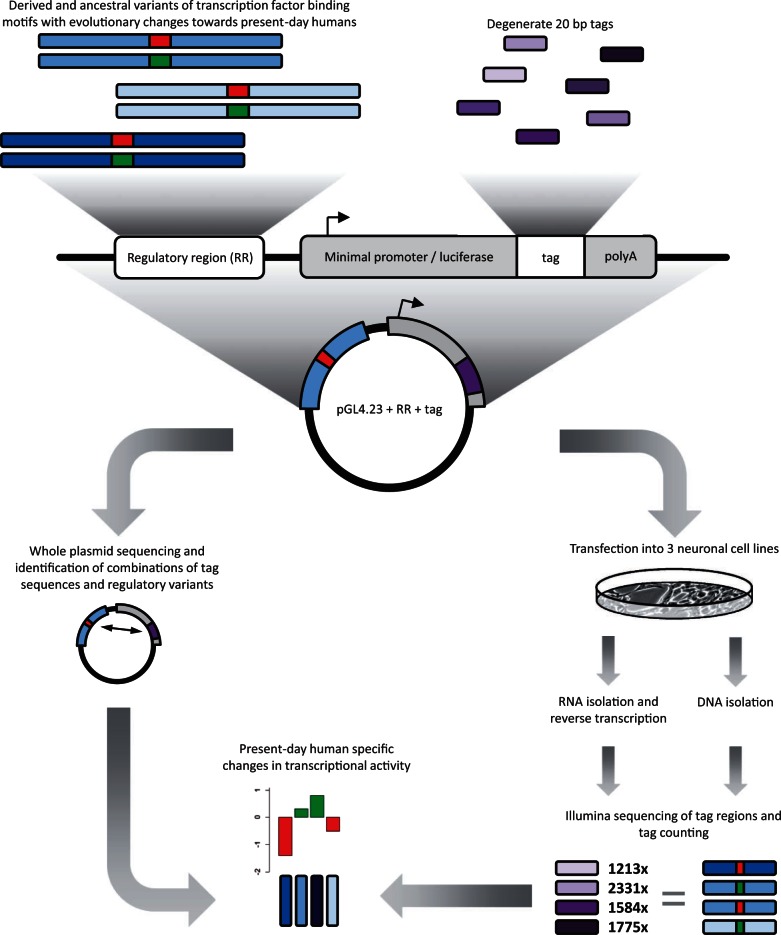
Overview of the RNA reporter assay. We synthesized 25 pairs of DNA sequences that carry ancestral (red) or derived (green) allelic variants of TFBSs and cloned them along with 20-nt-long tag sequences into a reporter vector (pGL4.23) containing a minimal promoter and the luciferase gene. The plasmids were sequenced to associate tags with TFBS variants, and plasmids prepared from mixed cultures of the 50 plasmids were transfected into SH-SY5Y cells, IMR-32 cells, and neural progenitor cells. RNA and plasmids were isolated after 24 h, and tag regions were amplified from the DNA and the reverse-transcribed RNA and sequenced. After correction for molar ratios of the plasmids in the cells, tag counts were compared for ancestral and derived versions of the TFBSs. Figure adopted from Patwardhan et al. (2012).

To investigate if these 25 TFBS alleles differ in their ability to drive transcription in neuronal cells, we cloned each TFBS variant as well as 25 nucleotides of flanking DNA on each side of the TFBSs upstream of a minimal promoter into reporter constructs (fig. 1) (Patwardhan et al. 2012). The promoter drives transcription from a reporter gene that carries 20-nt-long tags unique to each construct. As a positive control we prepared a similar reporter construct carrying a cytomegalovirus (CMV) enhancer and, as a negative control, a construct without any sequence inserted.

We pooled bacterial cultures carrying the 50 reporter plasmids as well as the positive and negative control plasmids and prepared DNA from the pools. These pools were transfected into two neuroblastoma cell lines (SH-SY5Y and IMR-32) and one neural progenitor cell line (Millipore ReNcell CX). Ten replicates of each transfection were performed, and 24 h after transfection RNA and DNA were isolated from the cells. From the RNA, transcripts from the reporter constructs were amplified after reverse transcription. From the DNA, the plasmids were amplified and sequenced in order to quantitate the molar ratios of the DNA pool in the cells. As a control for DNA carry-over into the RNA preparation, amplification from the RNA was performed without prior reverse transcription, and for each allelic form of the TFBSs the numbers of transcript observed when no reverse transcriptase had been added were subtracted from the numbers seen when cDNA had been synthesized. We then calculated the ratios of the transcripts observed for the derived TFBS variant over transcripts observed for the ancestral variant, and corrected for the ratios of the two corresponding DNA constructs extracted from the cells. Similarly, we calculated the ratios of transcripts observed for ancestral as well as derived TFBS variant over transcripts observed for the negative control and corrected for the ratios of the corresponding DNA constructs extracted from the cells (supplementary table S1, Supplementary Material online). Differences were corrected for multiple tests within the three cell lines and *P*-values below 0.05 were regarded as significant.

When we calculate the ratios of the transcripts produced from the ancestral and derived versions of the TFBSs relative to the negative control plasmid that lacks an inserted TFBS, we find that one of the TFBSs (chr2:9983591AG→A) results in significantly more transcripts in all three cell lines, three do so in two cell lines, and eight do so in one of the cell lines tested for at least one of the two variants (ancestral or derived). Thus, 12 of the TFBSs enhance transcription in the cell lines tested in their ancestral and/or derived variant. In contrast, only one of the TFBSs results in a significant reduction in numbers of transcripts (chr6:20212662T in SH-SY5Y cells) and thus may act as a repressor (supplementary table S1, Supplementary Material online).

**Fig. 2.**
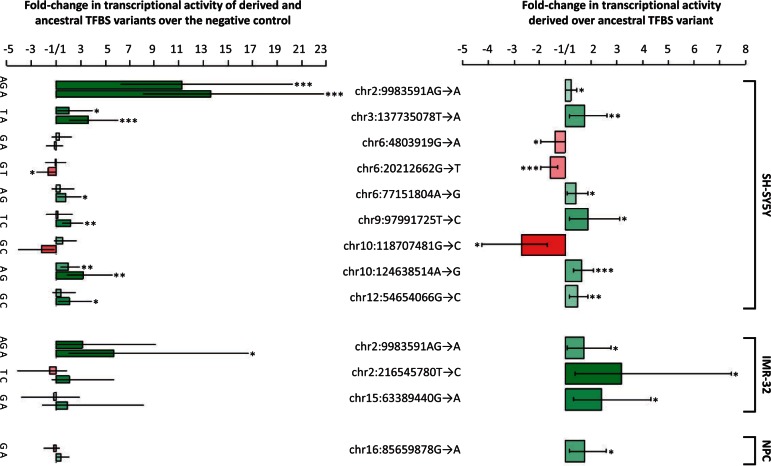
TFBSs for which ancestral variants seen in Neandertals and Denisovans and derived variants seen in present-day humans differ in their ability to generate transcripts. TFBSs with significant differences between ancestral and derived variant are shown (upper panel) as well as the transcriptional activity of the ancestral and derived variants relative to a negative control (lower panel). Note that the fold-change scale has no values between +1 and −1 and that +1.0 as well as −1.0 indicates no difference in transcriptional activity. NPC = neural progenitor cells. *P < 0.05, **P < 0.01, ***P < 0.001.

Six derived TFBS alleles show significant increases and three show significant decreases in transcriptional activity relative to their ancestral alleles in SH-SY5Y cells, while three derived TFBS alleles show significant increases in IMR-32 cells and one derived allele shows an increase in the neuronal progenitor cells (fig. 2, upper panel). Across all TFBSs, the ratios of the transcripts produced by the ancestral and derived variants are weakly but significantly correlated in the three cell lines (*R*_(NPC and IMR-32)_ = 0.26; *R*_(NPC and SH-SY5Y)_ = 0.30; *R*_(IMR-32 and SH-SY5Y)_ = 0.41) and slightly more so in the two neuroblastoma lines (*R* = 0.41). The average effect size of the differences between ancestral and derived variants is 1.8-fold. For seven of the TFBSs that show significant changes in transcript amounts between derived and ancestral variant, at least one variant produces significantly more or fewer transcripts than the negative control (fig. 2, lower panel). For the remaining five TFBSs, the numbers of transcripts produced by one variant is always higher and the other lower than the negative control, albeit not significantly so (fig. 2, lower panel). Some of these variants may lack activity in one form tested, or might exert larger effects if present together with cofactors.

In SH-SY5Y cells, the largest significant change in relative transcriptional activity is caused by the chr10:118707481G→C substitution, which reduces the expression of the derived allele to 38% of that of the ancestral allele (*P* = 0.01) and below the levels of the negative control (*P* = 0.06). This TFBS is expected to be bound by the transcription factor AP-1 and according to the “Transcribed Enhancer Atlas” (Andersson et al. 2014; Forrest et al. 2014) it may affect six genes (supplementary table S2, Supplementary Material online). Two of these, *HSPA12A* and *KIAA1598*, are highly expressed in the brain and encode a heat-shock protein and a protein involved in axonal induction, respectively. Transcripts from these two genes are less abundant in the frontal cortex of humans than of chimpanzees (Babbitt et al. 2010), as expected if the chr10:118707481G→C substitution is responsible for this expression difference. Other potential target genes are *PNLIP*, which encodes a pancreatic lipase that is essential for the efficient digestion of dietary fats (van Bennekum et al. 2000), and *PNLIPRP1* and *2*, which encode pancreatic lipase-related proteins. Interestingly, the PNLIP protein is one of only 87 proteins that carry a fixed amino acid change in modern humans relative to the Neandertal and Denisovan genomes (Prüfer et al. 2014). It is thus possible that both the regulation and activity of the pancreatic lipase have changed in modern humans.

Interestingly, another AP-1 binding site experienced a C→G substitution at position chr8:53608138. This substitution affects the same position in the TFBS but in the reciprocal direction to the chr10:118707481G→C substitution. In the SH-SY5Y cells, the chr8:53608138C→G substitution increases the transcriptional activity albeit not significantly (*P* = 0.09), that is, the effect is in the opposite direction to the reciprocal chr10:118707481G→C substitution (supplementary table S1, Supplementary Material online). The chr8:53608138C→G substitution is located in the first intron of *RB1CC1* and an enhancer transcript covering this site is correlated with the RNA transcript of *RB1CC1* across a variety of tissues and primary cells (Andersson et al. 2014; Forrest et al. 2014). *RB1CC1* encodes a transcription factor which is essential for maintaining adult neuronal stem cells in the subventricular zone of the cerebral cortex (Wang et al. 2013) and, like the PNLIP protein, the RB1CC1 protein is one of the 87 proteins that carry amino acid substitutions that are fixed in present-day humans but occur in the ancestral state in the Neandertal and Denisovan genomes. This change is located in one of 63 genomic regions for which there is evidence that they have been subject to positive selection in modern humans after their separation from archaic humans (Prüfer et al. 2014). It is thus tempting to speculate that the regulation of the *RB1CC1* gene and perhaps also the function of the encoded protein have changed and have been positively selected in modern humans.

At least two other TFBSs may affect the expression of transcription factors and can thus have multiple downstream effects: chr2:9983591G→A and chr10:124638514A→G. The derived version of the substitution chr2:9983591G→A is more active than the ancestral version in SH-SY5Y and IMR-32 cells. It may influence four genes: *KLF11*, *GRHL1*, *TAF1B*, which are transcription factors, and *ADAM17,* which encodes a protease that modifies a number of transcription factors and receptors (Beckmann et al. 1995; Asano et al. 1999; Wilanowski et al. 2002; Magrane and Consortium 2011). The genes *GRHL1*, *TAF1B*, and *ADAM17* all have higher transcript levels in frontal cortex of humans than of chimpanzees, whereas the expression of *KLF11* does not differ between the two species (Babbitt et al. 2010). It is thus tempting to speculate that this change may increase the expression of *GRHL1*, *TAF1B*, and *ADAM17* in humans which in turn may have numerous downstream effects. The substitution chr10:124638514A→G may affect the transcription of the gene *IKZF2*, which encodes a transcription factor involved in hematopoietic development (Hahm et al. 1998).

Several of the other changes that cause significant differences between the ancestral and derived alleles may warrant further analyses. For example, the TFBS affected by the substitution chr6:20212662G→T seems to actively repress transcription in SH-SY5Y cells because its derived variant causes the expression to be significantly lower than in the negative control lacking a TFBS (*P* = 0.03) (supplementary table S1, Supplementary Material online). It may regulate the gene *MBOAT1* which is involved in the development of the hand and foot (Dauwerse et al. 2007). The substitution chr6:4803919G→A may abrogate the expression of the gene *PPP1P3G* which is involved in glycogenesis (Zhang et al. 2014), and chr2:216545780T→C may increase the expression of the gene *FN1* which encodes fibronectin, a plasma protein involved in coagulation and wound healing (Lucena et al. 2007).

## Discussion

Until about 30,000 or 40,000 years ago, several hominin forms existed besides modern humans, the direct ancestors of present-day humans. All these hominins were similar in that they controlled fire, produced stone tools, and lived in social groups. However, from about 60,000 or 70,000 years ago, one of these hominin groups, “modern humans,” started to spread out of Africa and the Middle East and replaced other hominins. They eventually developed technology that changed rapidly and became progressively more complex. Modern humans also started producing figurative art and spread across vast expanses of water, with time colonizing essentially all habitable parts of the globe and becoming several orders of magnitudes more numerous than what other hominins had ever been. Neandertals and Denisovans never did these things although they existed for a longer time than modern humans have hitherto existed. It is tempting to speculate what this difference between modern and archaic humans had to do with cognitive differences between modern humans and other hominins (Klein 1999; Mellars 2006; Paabo 2014). However, it is still debated if these dramatic cultural and historical developments reflect any biological differences between modern and archaic humans or if they represent contingent cultural innovations unique to modern humans. It is thus a relevant question if genetic differences between present-day humans and Neandertals and Denisovans have consequences for the phenotype or not, particularly in the brain. Of special interest are differences that may influence gene expression as they may be particularly likely to affect phenotypic traits that differ between closely related populations and species (Wray et al. 2003).

To begin to address the question if DNA sequences that influence gene expression in neuronal tissues may differ between modern humans and Neandertals and Denisovans, we analyzed 25 TFBSs which carry derived variants at high frequency among present-day humans that do not occur in the Neandertal and Denisovan genomes and for which there are indications that they could be of functional significance. In 12 cases we find that the derived and ancestral alleles differ significantly in their ability to drive transcription in at least one of the three cell lines. Given that we tested only three cell lines and used a single reporter construct which may not allow each TFBS to exert its effects, the fact that we find differences between the two alleles for almost half of the TFBSs tested strongly suggests that a large fraction if not all of them affect the expression of target genes.

Although a difference in mRNA expression does not necessarily influence any biologically relevant phenotype (Khaitovich et al. 2004, 2005), there is some evidence that at least some of the changes analyzed here may have been positively selected. For example, the chr10:118707481G→C substitution which may regulate the gene *RB1CC1* is located in a region that has previously been suggested to be positively selected in modern humans after their separation from Neandertals and Denisovans (Prüfer et al. 2014). We thus suggest that the regulatory changes tested here as well as others that have recently risen to high frequency in humans are likely to have effects that have been of relevance for the evolution of the phenotypes that set modern humans apart from closely related archaic hominins.

## Methods

### Cloning of TFBSs into pGL4.23 Plasmid

The 50 TFBSs were from GeneArt Gene Synthesis. In each case, we centered the synthesized fragments on variable nucleotides and included 25 bp in each direction as well as *Kpn I* and *Nhe I* restriction sites. The TFBSs as well as a CMV enhancer (GenBank: U02451.1; nucleotides 18–431) were cloned into the pGL4.23 plasmid (Promega) and grown in *Escherichia coli DH5alpha* (New England Biolabs) and clones from each transformation were pooled and DNA extracted using the Qiaprep Spin Miniprep Kit. We synthesized a single-stranded oligonucleotide of 20 random bases (AAC CAT AGG ATT CGC GTC AGT CTA GAT AGN NNN NNN NNN NNN NNN NNN NGG CCG GCC GTC TGG CGT CTT GGC TAA TC) flanked on the 5’-end by an *Xba I* site and the sequence “TAG” and an *Fse I* site on the 3’-end (Invitrogen) as well as primer sites used to make the oligo double stranded by two cycles of polymerase chain reaction (PCR) (Thermo Scientific Phusion polymerase, annealing temperature 60 °C, primers AAC CAT AGG ATT CGC GTC AG and GAT TAG CCA AGA CGC CAG AC). The tags were cloned into the pGL4.23 plasmids carrying the TFBSs between the *Kpn I* and *Nhe I* sites, the CMV promoter, or no insert. DNA was extracted from individual clones using the Qiaprep Spin Miniprep Kit and colony PCR was performed (Thermo Scientific Phusion polymerase, annealing temperature 60 °C, primers TTT TTG TGT GAA TCG ATA GTA CTA ACA TAC GCT and GAG CTT CCA TTA TAT ACC CTC TAG TGT CTA AGC for regulatory regions, primers AGA TTC TCA TTA AGG CCA AGA AGG G and TCA CTG CAT TCT AGT TGT GGT TTG TCC for tag region). Plasmids that matched expected insert sizes for both amplifications were used to construct Illumina sequencing libraries (Meyer and Kircher 2010) and fully sequenced on an Illumina MiSeq (paired end 2 × (150 bp + 7 bp)) for sequence verification and association of the tags with TFBSs.

### Transfection of C*ell L*ines

Overnight cultures of clones with sequence-verified plasmids were set up (one per TFBS variant, three for chr1:26376528C, two for chr1:26376528T, a CMV positive control, a negative control plasmid). The next day, 5 ml of each (10 µl of CMV plasmid) was pooled and plasmids prepared (Invitrogen HiPure Plasmid Filter Midiprep). Neuronal cell lines (SH-SY5Y - ATCC; IMR-32 - ATCC; NPC - Millipore) were cultured according to the protocols provided by the vendors and transfected with 2 µg of the plasmid pool, using a Lonza Nucleofector 2b device and the Lonza Amaxa Nucleofector kits (V, L, and Mouse NSC, respectively) and recommended nucleofection programs (A-030, C-005, and A-033, respectively). Twenty-four hours after transection, cells were harvested, pellets were flash frozen in liquid nitrogen, and stored at −80 °C.

### Nucleic Acid Isolation and Sequencing

Cell pellets were resuspended in 350 µl Qiagen RLT buffer and homogenized using QiaShredder columns. Seventy microliters were used for plasmid purification (Qiaquick PCR Purification Kit) and the remaining 270 µl for RNA purification (Qiagen RNeasy Mini Kit). After incubation with 20 µl of DNase I solution (Qiagen RNase free DNase Kit) for 60 min at room temperature, mRNA was isolated (Thermo Scientific MagJET mRNA Enrichment Kit) and split into an RTminus aliquot and an aliquot that was reverse transcribed (Thermo Scientific DyNAmo cDNA Synthesis Kit).

Both RNA samples and the plasmid samples were subjected to quantitative PCR (qPCR; New England Biolabs DyNAmo Flash SYBR Green qPCR Kit) using primers amplifying the tag region as described (Meyer and Kircher 2010). Reactions were stopped one cycle after the steepest increase of the qPCR curve of the RTplus reaction and indexing PCR for sequencing was done as described (Meyer and Kircher 2010), while taking care to stop also the indexing reactions when the RTplus curve passed the inflection point in order to not saturate the PCRs of RTplus samples.

After sequencing (Illumina MiSeq, paired end 2 × (150 bp + 7 bp)) and base calling (Bustard, Illumina), overlapping paired-end reads were merged using *leeHom* (Renaud et al. 2014), tags associated with TFBS alleles using *deML* (Renaud et al. 2015), and the number of occurrences of each tag counted (supplementary table S3, Supplementary Material online).

### Analyses of Transcript *N*umbers

All analyses were implemented in R (version 3.0.3) (R Core Team 2014) using Tinn-R (Faria et al. 2013). Replicates were excluded from the analysis when count numbers for a variant were unusually high in the RTminus sample or unusually low in the RTplus sample compared with all other replicates (as indicated in supplementary table S3 and fig. S1, Supplementary Material online). For any given replicate of a TFBS, the normalized ratio of derived (*d*) over ancestral (*a*) tag counts were calculated as follows:

$$\frac{\text{d}}{a}=\text{ln}\left(\frac{\frac{\text{d}\;\text{RTplus}-\text{d}\;\text{RTminus}}{\text{a}\;\text{RTplus}-\text{a}\;\text{RTminus}}}{\frac{\text{d}\;\text{DNA}\;\text{input}}{\text{a}\;\text{DNA}\;\text{input}}}\right)$$

Ratios of tags expressed from TFBSs over the negative control were similarly calculated (supplementary table S1, Supplementary Material online). One sample *t*-tests (R: t.test*(x)*) were used to assess whether the normalized ratios of tag counts differ from 0 (i.e., no difference in transcript numbers). Similarly, *t*-tests were used to assess whether the transcript numbers of a given TFBS variant differ from that of the negative control. *P* values were corrected for multiple testing within each cell line (Benjamini and Hochberg 1995) (R: p.adjust(method=”BH”)) and *P* values smaller than 0.05 were regarded as significant. Correlations of average expression ratios among cell lines were calculated using the R function cor(method=”pearson”).

### Public Data Sets

Transcription start sites were defined according to “RefSeq release 63” (Pruitt et al. 2009) using the function *findClosestGene* in the R package “ACME” (Scacheri et al. 2006) and enhancer-transcription start site associations were from the “Transcribed Enhancer Atlas database” (Andersson et al. 2014; Forrest et al. 2014). All transcription factors that bind to the TFBSs according to JASPAR (supplementary table S2, Supplementary Material online) are expressed in the human brain according to Human Body Map 2.0 data set and BioGPS (Wu et al. 2009, 2013; Derrien et al. 2012).

## Supplementary Material

Supplementary figure S1 and tables S1–S3 are available at *Molecular Biology and Evolution* online (http://www.mbe.oxfordjournals.org/).

Supplementary Data
