# Supplementary material for: Functional Analyses of Transcription Factor Binding Sites that Differ between Present-Day and Archaic Humans
Source: Mol Biol Evol. 2015 Oct 9;33(2):316–22. doi: 10.1093/molbev/msv215 (PMC4866544; doi:10.1093/molbev/msv215)
Supplement: Supplementary Data [file supp_33_2_316__index.html]

Functional Analyses of Transcription Factor Binding Sites that Differ between Present-Day and Archaic Humans — Functional Analyses of Transcription Factor Binding Sites that Differ between Present-Day and Archaic Humans — Supplementary Data 

# Functional Analyses of Transcription Factor Binding Sites that Differ between Present-Day and Archaic Humans

## Supplementary Data

files

- Supplementary Data - xlsx file
- Supplementary Data - xlsx file
- Supplementary Data - pdf file
