# Supplementary figures and images for: Functional Analyses of Transcription Factor Binding Sites that Differ between Present-Day and Archaic Humans
Source: Mol Biol Evol. 2015 Oct 9;33(2):316–22. doi: 10.1093/molbev/msv215 (PMC4866544; doi:10.1093/molbev/msv215)

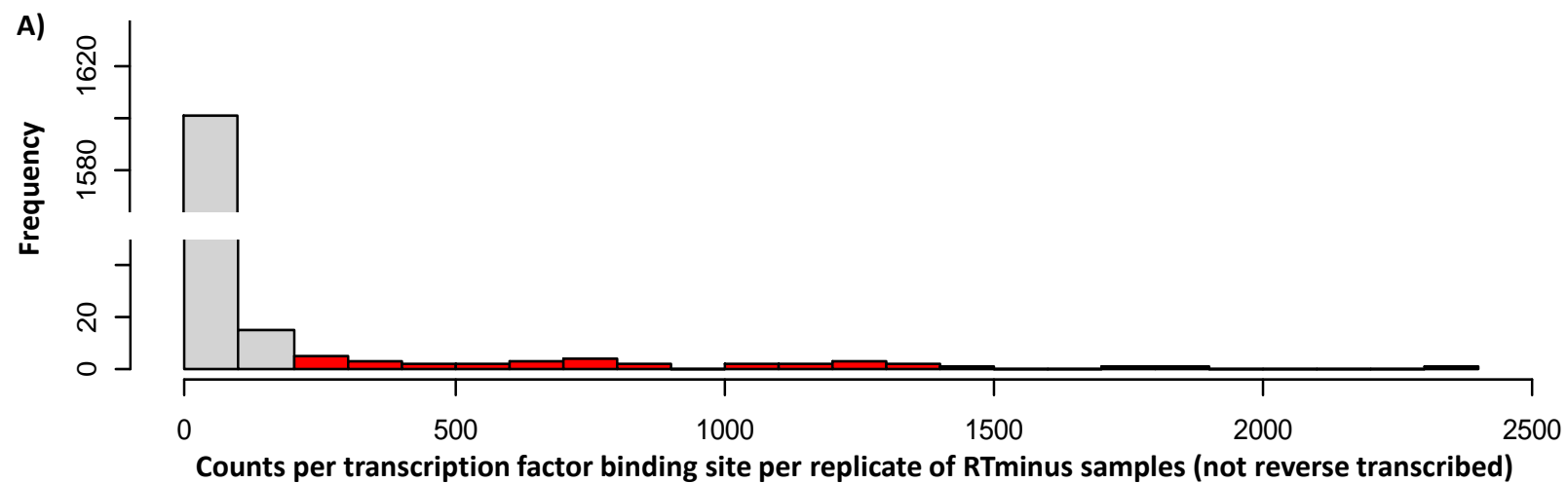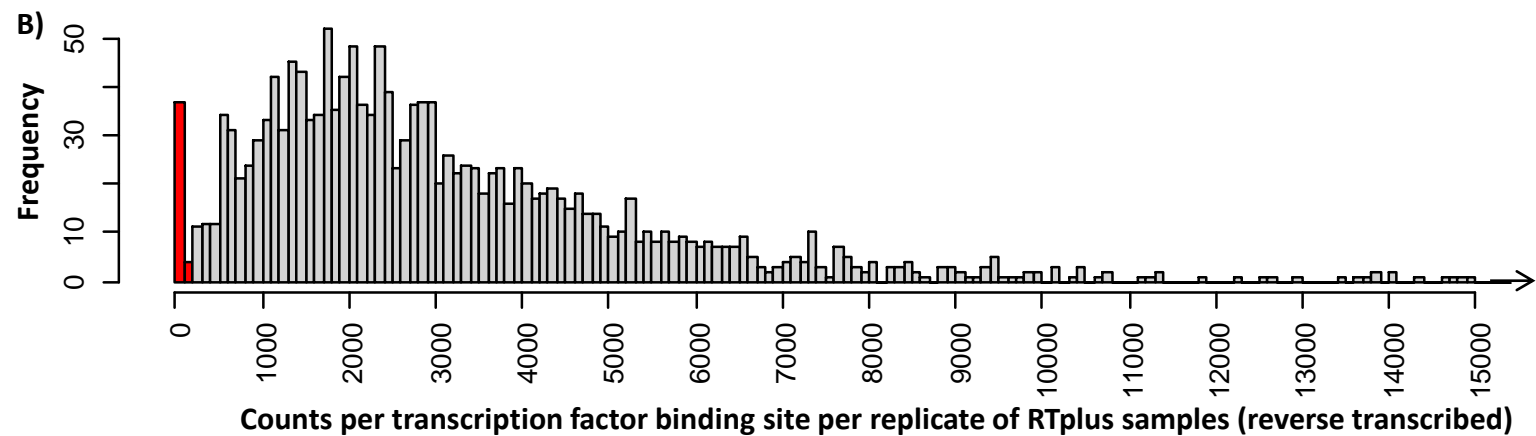

Supplement: Supplementary Data [file supp_msv215_Supplementary_Figure_1.pdf]
